# Dual Role of Reactive Oxygen Species in Muscle Function: Can Antioxidant Dietary Supplements Counteract Age-Related Sarcopenia?

**DOI:** 10.3390/ijms20153815

**Published:** 2019-08-05

**Authors:** Simona Damiano, Espedita Muscariello, Giuliana La Rosa, Martina Di Maro, Paolo Mondola, Mariarosaria Santillo

**Affiliations:** Dipartimento di Medicina Clinica e Chirurgia, Università di Napoli “Federico II”, Via S. Pansini, 5, 80131 Naples, Italy

**Keywords:** sarcopenia, reactive oxygen species, redox signaling, antioxidant supplementation, exercise

## Abstract

Sarcopenia is characterized by the progressive loss of skeletal muscle mass and strength. In older people, malnutrition and physical inactivity are often associated with sarcopenia, and, therefore, dietary interventions and exercise must be considered to prevent, delay, or treat it. Among the pathophysiological mechanisms leading to sarcopenia, a key role is played by an increase in reactive oxygen and nitrogen species (ROS/RNS) levels and a decrease in enzymatic antioxidant protection leading to oxidative stress. Many studies have evaluated, in addition to the effects of exercise, the effects of antioxidant dietary supplements in limiting age-related muscle mass and performance, but the data which have been reported are conflicting. In skeletal muscle, ROS/RNS have a dual function: at low levels they increase muscle force and adaptation to exercise, while at high levels they lead to a decline of muscle performance. Controversial results obtained with antioxidant supplementation in older persons could in part reflect the lack of univocal effects of ROS on muscle mass and function. The purpose of this review is to examine the molecular mechanisms underlying the dual effects of ROS in skeletal muscle function and the analysis of literature data on dietary antioxidant supplementation associated with exercise in normal and sarcopenic subjects.

## 1. Age-Related Sarcopenia 

Aging is characterized by a progressive decline in muscle mass and strength. In 1988 Irwin Rosenberg proposed the term sarcopenia (from the Greek “sarx” = flesh and “penia” = loss) to describe muscle size decrease that occurs in the elderly [1].

Roubenoff tried to differentiate sarcopenia from other processes leading to muscle mass loss, such as wasting and cachexia [2]. Wasting was considered an unintentional weight loss, including loss of both fat and lean body mass, due to inadequate caloric intake. Cachexia, on the other hand, was defined as “the loss of fat free mass with no significant weight loss“ as a result of hypermetabolism and hypercatabolism mediated by cytokines. Conversely, sarcopenia was regarded as a process that could take place even without malnutrition or disease and in this sense could be considered a natural process in aging. The currently most used definition in clinical practice and diagnostic criteria consensus for age-related sarcopenia has been developed by the European Working Group on Sarcopenia in Older People (EWGSOP) [3], which claims that a subject with low muscle strength and low muscle mass or quality must be diagnosed with sarcopenia. Hence, sarcopenia can occur either acutely or chronically. Diagnosis of sarcopenia relies on combined measurement of (1) muscle mass, which is assessed for example by dual energy X-ray absorptiometry (DXA) or bioelectrical impedance analysis (BIA); (2) muscle strength, assessed by functional tests like grip strength; and (3) physical performance, including assessment of mobility, strength, and balance [4].

Sarcopenia can be considered a multifactorial event which is characterized by inflammation, oxidative stress, motor neuron loss, and changing of endocrine function [5]. Loss of strength and mobility, together with balance disorders induced by sarcopenia, increase the rate of falls and fractures in old age, leading to immobilization, which in turn contributes to worsening of sarcopenia. In addition, old age is often associated with appetite loss, protein-energy malnutrition, and weight loss, which concur with sarcopenia. Therefore, an adequate nutrient intake is recommended in sarcopenic patients to preserve muscle mass. Often physical inactivity and malnutrition reinforce each other; indeed, reduced mobility leads to a decline in nutrition capability, worsening malnutrition and sarcopenia. However, all these factors contribute to skeletal muscle atrophy and weakness, accompanied by disability increase, frailty, and life quality impairment [5].

Several studies have focused on the pathophysiology of age-related sarcopenia; nonetheless, the biological mechanisms underlying decline in muscle strength and mass with age are not completely understood. Loss in muscle mass and impairment of muscle force has been associated with the disruption of excitation–contraction coupling. In single human skeletal myocytes obtained by needle biopsy of the vastus lateralis, a significant reduction of dihydropyridine (DHP)-sensitive Ca^2+^ currents have been recorded in fibers from old people compared to those in young people. Moreover, a reduced peak of Ca^2+^ transient and a decrease in the voltage-/Ca^2+^-dependent Ca^2+^ release ratio has been registered in old fibers compared to young ones, suggesting that the decrease of Ca^2+^ available for mechanical responses in aged skeletal muscle is due to DHP receptor (DHPR)-ryanodine receptor (RyR) uncoupling [6]. Similar results have been obtained in experiments conducted in mice [7]. Age-associated muscle mass and strength decline may also be explained by motor unit remodeling, which has been observed in old mice, in which denervated muscle fibers are reinnervated by axonal sprouting of adjacent motor units [8]. Moreover, fibers from aged rats contract more slowly than those from young rats; in an in vitro motility assay with isolated soleus muscle fibers an age-related alteration in myosin with a decrease of the maximum shortening velocity has been evidenced [9]. A replacement of muscle mass by fat and connective tissue with aging results in a gradual decrease of muscle size/volume. Moreover, muscle biopsies from old and young subjects have shown that number and size of muscle fibers, mainly of type II (fast-twitch) are reduced in the elderly [10]. Indeed, a loss of muscle mass explains only in part muscle decline, and other possible mechanisms are related to alterations in muscle fiber quality and changes in fiber type [11]. It has been also hypothesized that a reduction and/or dysregulation of satellite cells involved in skeletal muscle regeneration may contribute to the loss of skeletal muscle mass observed in aging [12]. Among the mechanisms underlying sarcopenia, an emerging role is being played by endoplasmic reticulum (ER) stress due to accumulation of unfolded or misfolded proteins within the ER and its adaptive responses involving reactive oxygen species (ROS) signaling [13]. Finally, recent studies on the role of gut microbiota in inflammatory diseases indicate a relevant contribution of gut microbial changes and activity that occur with aging to the types of inflammatory molecules present in the environment surrounding muscles [14].

The molecular mechanisms leading to sarcopenia are complex and have not been completely clarified. Redox signaling and oxidative damage are among the most accepted mechanisms underlying the decline of muscle mass and strength with aging and they will therefore be discussed in more detail in separate sections of this review.

## 2. Endogenous Sources of Reactive Oxygen Species and Antioxidant Systems

ROS are generated through various pathways; one of the main sites of ROS production is the mitochondrial electron transport chain, where the transfer of a single electron to molecular oxygen gives rise to a monovalent reduction of oxygen, which leads to the formation of superoxide ions. The superoxide production can also occur enzymatically through NADPH oxidase enzymes or the xanthine/xanthine oxidase system. NADPH oxidase was discovered first in phagocytes, where it generates high levels of superoxide as a microbicidal mechanism in host defense [15]. NADPH oxidase is an enzymatic complex consisting of various cytosolic units (p40-, p47-, and p67phox), and the membrane-anchored components p22phox and cytochrome b558 (gp91phox); the latter is the site of the catalytic activity of the complex. The activation of the complex also requires the involvement of small GTP binding proteins Rap1A and Rac. In mammalians there are seven genes encoding distinct catalytic subunits, namely, NOXs 1-5 and DUOX1-2 [16]. Xanthine oxidase enzyme catalyzes the hydroxylation of hypoxanthine to xanthine and xanthine to uric acid. In both steps, molecular oxygen is reduced, forming the superoxide anion [17].

Among the antioxidant enzymes, superoxide dismutases are responsible for the dismutation of O_2_^−^ in molecular oxygen and hydrogen peroxide [18,19], which is converted into water by catalase or glutathione-peroxidase. There are three SOD isoenzymes, namely, the cytosolic dimeric Cu,Zn SOD (SOD1) [20], the mitochondrial manganese-containing SOD (MnSOD or SOD2) [21], and the tetrameric extracellular CuZn SOD (EcSOD or SOD3) [22].

In the reaction catalyzed by glutathione-peroxidase, glutathione is oxidized to glutathione disulfate, which can be converted to glutathione by glutathione reductase in a “NADPH-consuming” process (NADPH → NADP^+^) [23]. Catalase is a high molecular weight tetrameric enzyme containing porphyrin in the active site [24]. In the presence of transition metal ions (e.g., Fe^2+^\^3+^, Cu^+^\^2+^), hydrogen peroxide produces the highly reactive oxygen species hydroxyl radical (OH**^.^**) and hydroxyl ion (OH^−^) according to the Fenton reaction [25].

Endoplasmic reticulum (ER) is involved in the control of the redox state of the cells as well [26]. The accumulation of unfolded or misfolded proteins within the ER leads to ER stress and to the activation of the signaling pathways of the unfolded protein response (UPR) as adaptive response. Aiming to re-establish ER proteostasis, the UPR pathway reduces ER protein load and enhances ER quality control and autophagy. The adaptive response of the UPR pathway induces antioxidant gene transcription through the activation of nuclear factor E2-related factor 2 (Nrf 2). However, the UPR pathway can even enhance ROS production; indeed, the increased protein folding during ER stress causes the peroxide levels to rise through the pancreatic ER kinase (PERK)/C/EBP homologous protein (CHOP) pathway, thus enhancing the expression of Ero1, encoding for an ER peroxidase. This pathway leads to oxidative stress, and, eventually, apoptosis [27].

The levels of ROS/reactive nitrogen species (RNS) inside the cells are strictly controlled by the balance between the rate of synthesis by ROS/RNS generating systems and the rate of removal through the non-enzymatic and enzymatic antioxidant systems. An accurate control of ROS levels guarantees the maintenance of physiological levels of ROS necessary for cell functions. Excessive ROS/RNS production or the impairment of antioxidant status may disturb the cellular redox balance, inducing oxidative stress in cells or tissues.

## 3. The Role of Reactive and Nitrogen Species in Muscle Functions

Many studies suggest that high levels of free radicals can damage biological molecules while low levels play physiological roles such as regulation of cell signaling [28,29,30,31,32,33,34,35,36]. ROS, including mainly superoxide anions and hydrogen peroxide and hydroxyl radicals, are continuously produced by muscle cells in resting condition, and their levels increase during contraction modulating force production [37]. However, while at low levels a ROS increase produces an enhancement in the development of muscle force up to a maximum peak, a further ROS increase induces a dramatic decline in the force [38]. Moreover, intense and prolonged exercise has often been associated with increase of ROS production and oxidative damage to cellular constituents.

ROS are produced in muscle cells by various sources in different cell compartments. Mitochondria are an important site of ROS production, and during exercise, ROS increases due to high oxygen consumption by increased mitochondrial activity [39,40]. Another source of ROS in skeletal muscle is NOX enzymes [41]. NOX1, NOX2, and NOX4 are three NOX isoforms expressed in skeletal muscle [42]. NOXs are located in the sarcoplasmic reticulum (SR), transverse tubule, and plasma membrane; in particular, the superoxide anion generated by NOX2 in the SR can stimulate the correct release of calcium from intracellular stores through the oxidation of RyR1 [43]. Thus, this enzyme plays an important functional role in excitation contraction coupling. Although mitochondria have long been considered the principal site of muscle ROS production [35], much evidence suggests that NOXs are the main source of ROS induced by skeletal muscle contractions. Indeed, during contractions, the increase in cytosolic ROS comes first and is greater than the rise in mitochondrial ROS [44].

ROS-mediated ROS release due to crosstalk between NOXs and mitochondria has been described for different cells. The uncoupling of electron transfer can result from oxidation of mitochondrial electron transfer chain complexes [45]. Moreover, NOX-derived ROS can induce mitochondrial ROS production through the opening of mitochondrial ATP-sensitive K+ channels (mito-KATP) [46] and consequent potassium influx into the matrix that reduces the mitochondrial membrane potential, opening permeability transition pores. Another mechanism that can explain the crosstalk between NOXs and mitochondrial ROS production is the rise of intracellular Ca^2+^ levels by NOX-derived ROS, which, increasing mitochondrial Ca^2+^ load, induce ROS production by these organelles [47]. The existence of NOX-mitochondria redox crosstalk has also been demonstrated insmooth and cardiac muscle cells. Although there is no direct evidence of the existence of ROS-mediated ROS release in skeletal muscle cells, it is plausible that these mechanisms are also active in these cell types.

Another striking example of ROS-mediated ROS release is the crosstalk between ER and mitochondria. ER oxidative stress can be transmitted to mitochondria through Ca^++^ influx. Across mitochondrial-associated ER membranes (MAMs), Ca^++^ ions and other metabolites are transferred to mitochondria, leading to the opening of permeability transition pores and increased mitochondrial ROS levels [26].

RNS as well as ROS may be messenger molecules that activate muscle adaptive responses through the induction of redox-sensitive signaling to maintain cellular oxidant-antioxidant homeostasis during exercise. RNS arise from several sources and the levels increase with contractile activity. Nitric oxide (NO) is formed from L-arginine in a reaction catalyzed by the nitric oxide synthase (NOS) enzyme. In skeletal muscle there are all the different isoforms of this enzyme: nNOS, eNOS, and iNOS. A calmodulin (CaM)-binding domain is present in nNOS and eNOS, and, therefore, these Ca^2+^sensitive isoforms are responsive to contractile activity [48]. Moreover, NO generated by NOSs readily reacts with superoxide to form peroxynitrite (NO^+^ O_2_^−^/ONOO^−^), which decreases the bioavailability of NO and superoxide, modifying the redox balance in the myocyte [49].

Regular physical exercise modulates ROS in a bell-shaped hormesis curve due to the activation by ROS of adaptive response resulting in increasing activity of repair enzymes and low degree of oxidative stress [50]. Overall, it seems that with the exception of high intensity and long duration exercise, physical activity cannot result in harmful oxidative damage.

## 4. ROS-Mediated Mechanisms in the Development of Age-Related Sarcopenia

Cumulative damage to skeletal muscle and nerve cells in sarcopenia may result from oxidative stress. Oxidative stress causes damage in many tissues, including the loss of muscle mass and strength, which is associated with impairment of neurotransmitter release and neuronal degeneration.

Sarcopenia could be caused by an increase of endogenous ROS formation in skeletal muscle but the source of ROS in sarcopenic muscle is still relatively unknown; however, an age-associated increase of ROS levels in muscle mass, as a consequence of an upregulation of NOX2 enzyme, has been reported [51]. Moreover, a study by Sullivan-Gunn and Lewandowski [52] has highlighted the role of NOX2 enzyme in a healthy mouse model of aging, suggesting that elevated levels of H_2_O_2_ from NOX2, as well as the lack of antioxidant protection from catalase and glutathione peroxidase (GPx), carry out a key role in the onset of sarcopenia. The lack of SOD1 also causes a reduction of skeletal muscle mass, impairment of neurotransmitter release, and neuronal degeneration in mice [53].

O_2_^−^ radicals induce neuromuscular degeneration and mitochondrial dysfunction in SOD1-deficient mice [54]; moreover, the reduction of cellular antioxidant capacity by disruption of the SOD1 gene in mice (*Sod1*^−/−^) causes severe oxidative stress and oxidative damage associated with an acceleration of age-related loss of skeletal muscle mass accompanied by neuromuscular junction (NMJ) morphologic changes, increased denervation, and an elevated production of superoxide and hydrogen peroxide by muscle mitochondria [54]. Elevated ROS have been shown to interfere with synaptic vesicle axonal transport and formation of new vesicles in the trans-Golgi network [55]. This, in turn, may lead to the accumulation of fewer synaptic vesicles at NMJs, resulting in reduced neurotransmitter release [56]. In addition, *Sod1* gene ablation in adult mice causes physiological changes at the NMJ, similar to that occurring in old wild types [57]. Moreover, the increase of cytosolic oxidative stress caused either by the deletion of SOD1 (*Sod1*^−/−^ mice) or by introduction of mutations of SOD1, (e.g., the SOD1^G93A^ amyotrophic lateral sclerosis mutant mouse model) increases ROS levels, causing muscle atrophy and weakness in mice which are phenotypically similar to muscle changes observed in older animals [54,58].

The use of SOD1^G93A^ and other SOD1 mutant models to decipher the mechanistic aspects of oxidative stress in muscle atrophy could be confounded by the toxic gain of function that results in the formation of SOD1 protein aggregates [59]. Indeed, recently, it has been shown that a gain of function of the mutated SOD1^G93A^ could be associated with an increase of oxidative stress, intracellular calcium concentration, and proapoptotic effect in both human neuroblastoma SK-N-BE and mouse motor-neuron-like NSC-34 cells. These effects are carried out through an activation of extracellular signal regulated kinases (ERK) 1–2, serine threonine kinase (Akt), and intracellular calcium levels mediated by the activation of muscarinic M1 receptor [28]. Systemic administration of endogenous nitric oxide donor *S*-nitrosoglutathione enhances extracellular SOD3 expression and the antioxidant activity protecting structural and functional integrity of skeletal muscle [60]. However, it is noteworthy to underline that SOD1 is secreted as well [61], and, therefore, the elevated extracellular oxygen radical, associated with sarcopenia, could also be scavenged by cytosolic SOD1.

ER stress and UPR response also play an important role in age-related sarcopenia [13]. ER stress can directly impact muscle mass since sustained ER stress leads to cell death of muscle cells [62], which is mediated by increased ER ROS. ER stress can also inhibit rapamycin complex 1 (mTORC 1) that mediates the response to anabolic stimulus of nutrients and contractile activity, thus inducing anabolic resistance and reduced regenerative potential of skeletal muscle observed during aging. Moreover, it has been shown that in the chronic kidney disease uremic toxin-accumulated sarcopenia model, ER stress and UPR pathways account for the inhibition of myoblast differentiation and myotubular atrophy induction through the activation of a ROS-eIF2α axis [63].

Aging is also characterized by mitochondrial dysfunction in skeletal muscle with accumulation of mitochondrial damage and oxidative stress [64]. Mitochondrial dynamics are controlled by fusion and fission proteins. Mitofusin 1 and 2 (Mnf 1 and 2) are involved in the outer mitochondrial membrane fusion while optic atrophy 1 (OPA1) mediates fusion of the inner mitochondrial membrane. Aging is associated with a progressive reduction in Mnf2, and Mfn2 deficiency in mouse skeletal muscle reduces mitophagy, leading to the accumulation of damaged mitochondria [65]. On the other hand, OPA1, which is also a sensor of physical activity, is downregulated during aging-related sarcopenia. Interestingly, in adult mice, acute, muscle-specific deletion of OPA1 leads to ER stress, which through UPR pathways, ROS, and FoxOs induces a catabolic program, muscle loss, and systemic inflammation [66].

In conclusion, age-related ROS overproduction generates oxidative damage of muscle but it also plays a role in regulating intracellular signal transduction pathways that are directly or indirectly involved in skeletal muscle atrophy, motoneuronal degeneration, and impairment of muscle contractility.

## 5. Redox Signaling in Exercise Adaptation in Age-Related Sarcopenia

Muscle has meaningful plastic properties. Indeed, regular physical exercise ameliorates skeletal muscle performance along with other multiple body functions. During exercise the great oxygen flux required for ATP production leads to ROS generation at different rates and from different sources depending on type, intensity, and duration of exercise. ROS generated during exercise regulate signal transduction pathways responsible for muscle remodeling and for the adaptive response necessary to limit oxidative stress.

Nuclear factor (NF) κB, mitogen-activated protein kinases (MAPKs), and peroxisome proliferator-activated receptor γ co-activator 1 α (PGC1α) are among the main redox-sensitive pathways activated during muscle activity involved in the adaptive response to oxidative stress [67].

NFκB, induced by hydrogen peroxide and pro-inflammatory cytokines, increases the expression of proteins and enzymes that require consensus binding of κB, including SOD2, glutamyl-cysteine synthetase (GCS), iNOS, and cyclooxygenase 2 (COX2), among others [68].

MAPK pathways, comprising c-Jun N-terminal kinases (JNK), ERK 1–2, and p38 MAPK, are activated by a variety of physiological events associated with exercise like ROS, hormones, calcium influx, and neural or mechanical stimuli [69]. Through p38 MAPK activation/phosphorylation, ROS increases glucose uptake by muscle cells during exercise [70].

PGC1α plays a pivotal role in the regulation of mitochondria biogenesis, antioxidant enzyme expression, and regulation of anti-inflammatory cytokine expression [67]. Moreover, PGC1α promotes mitochondrial oxidative metabolism [71] and plays a role in fiber-type specificity inducing slow phenotype specification [72]. PGC1α is regulated at transcriptional and post-transcriptional levels by pathways activated during muscle contraction like AMP-activated protein kinase (AMPK), sirtuin 1 (SIRT1), protein kinase C, changes in intracellular Ca^2+^ concentration, p38 MAPK, NO, ROS, and hypoxia-inducible factor-1 (HIF-1) [73]. An important anabolic pathway inducing protein synthesis involves activation of the phosphatidylinositol 3-kinase (PI3K)/Akt, which stimulates mammalian target of rapamycin (mTOR), which is essential for muscle growth during development and regeneration; this pathway has a role also in the regulation of muscle mass which strictly depends on protein synthesis [74]. In most cases, the effects of ROS on these signaling molecules differ in dependence of the levels. For example, JNK phosphorylation levels depend on the relative activity of specific kinases and phosphatases. Low levels of ROS induce JNK phosphorylation without affecting phosphatase, leading to a transient activation of JNK, while higher ROS levels may activate the JNK pathway and inactivate phosphatases, resulting in a prolonged activation of JNK [75]. In addition, ROS activate the PI3K/Akt pathway, either by directly activating PI3K or inactivating phosphatase and tensin homolog (PTEN) which inhibits the activation of Akt through cysteine residues oxidation [76]. At lower levels, ROS also oxidize the disulfide bridges in Akt leading to a short-term activation of Akt signaling [77].

Muscle adaptation to different training conditions is associated with the activation of different pathways. The main muscle adaptive response to non-exhaustive endurance training is mitochondria biogenesis, which increases muscle oxidative capability [78]. The underlined mechanism relies on the production of reactive oxygen species like ubisemiquinone, NO, superoxide, and H_2_O_2_ [79,80].

A key modulator of mitochondria biogenesis is PGC1α, which guarantees the balance between the production and scavenging of reactive oxygen species by regulating both mitochondrial biogenesis and the expression of antioxidant enzymes like SOD1, GPx, and catalase [81]. Unlike endurance training, resistance training induces skeletal muscle hypertrophy. Indeed, resistance training is associated with an increase in protein biosynthesis with respect to protein breakdown. ROS produced during resistance exercise activate multiple pathways, including the insulin/IGF-1-IP3K, MAPKs, and Ca-calmodulin pathways [82] involved in protein biosynthesis.

Finally, short-term anaerobic exercise, such as sprinting, is associated with high levels of ROS and oxidative stress. During sprinting exercise, the main ROS sources are NOXs [83] and the xanthine/xanthine oxidase system [84], while mitochondria play only a minor role. Additionally, exhaustive endurance and resistance exercise are related to increased levels of skeletal muscle ROS, oxidative stress, and cortisol, leading to transitory immunosuppression [85]. In the elderly, protein anabolic pathways are reduced [86] while protein catabolic pathways are activated [87], contributing to muscle atrophy. In particular, with aging a reduction in the mitochondrial protein synthesis rate in muscle is correlated with a decrease in mitochondrial enzyme activity and oxidative capability [88]. In addition, a significant decline in mitochondria biogenesis, lower levels of testosterone, and a PGC1α, Akt, and mTOR expression decrease contribute to the loss of muscle mass and strength, which are hallmarks of sarcopenia [89,90]. Much evidence highlights the role of reactive oxygen species in age-related neuromuscular deficit [91]. Impaired mitochondrial electron transport chain has been involved in the increase of ROS levels in aged skeletal muscle [92]; moreover, in aging, skeletal muscles produce higher levels of ROS during an acute bout of exercise, while chronic exercise has a protective effect against oxidative damage [93]. In addition, physical inactivity increases the levels of oxidative stress contributing to the onset of sarcopenia [94].

Much evidence highlights the positive role of exercise in elderly skeletal muscle. Elderly subjects who regularly exercise show oxidative stress levels comparable to that of younger individuals who do not perform physical activity [95].

Both redox-sensitive mitochondria biogenesis and PGC1α levels are increased by exercise training and increased expression of PGC1α in old mice is associated with high mitochondria biogenesis and lower oxidative stress, inflammation, and apoptosis [89].

Overall, ROS/RNS signaling plays a pivotal role in skeletal muscle contractile function, hypertrophy, mitochondrial biogenesis, and glucose uptake as an adaptive response to exercise. Increasing knowledge of redox signaling involved in responses to exercise will lead to the development of new approaches to regulate muscle metabolism and function to prevent loss of muscle mass and performance in age-related diseases.

## 6. Dietary Intervention in Age-Related Sarcopenia

It is well known that old age is often associated with appetite loss, which contributes to decreased food intake, protein-energy malnutrition, and weight loss. Therefore, it is important to evaluate different dietary interventions to assure an adequate nutrient intake in sarcopenic patients to preserve muscle mass.

In the elderly, muscle protein homeostasis is impaired because of a reduced synthesis and increased rate of degradation. In addition, reduction in muscle mass is facilitated by physical inactivity and decreased dietary protein intake [96]. Several authors suggest the consumption of good sources of proteins low in fat, including lean meat, poultry, and fish, and high protein intake (1.2–1.4 g/Kg/die) [97] for the treatment of sarcopenia. In vitro experiments, performed in cardiomyoblast cell lines, have shown that serum from vegan subjects induces oxidative stress and cell death compared to vegetarian and omnivorous sera [98], suggesting a mechanistic link between deficient protein intake, oxidative stress, and loss of muscle mass.

The list of natural compounds with antioxidant activity is very long. However, we have focused our attention on the most known nutritional antioxidants, including L-ascorbic acid (vitamin C), tocopherols (vitamin E), carotenoids, flavonoids, and polyphenols [99,100,101,102,103,104,105], whose function on muscle have been more extensively studied.

Vitamin C is the primary water-soluble and non-enzymatic antioxidant in plasma and tissues. Humans, unlike most mammals and other animals, do not have the ability to synthesize vitamin C due to lack of the last enzyme in the biosynthetic process, and it must therefore be obtained by dietary intake. The principal sources of vitamin C are kiwifruit, strawberries, broccoli, kale, tomatoes, and sweet red pepper [106]. Important enzymatic reactions requiring vitamin C as an essential cofactor are the biosynthesis of collagen, carnitine, and neuropeptides, and the regulation of gene expression [107]. Cohort studies also indicate that higher vitamin C status, assessed by measuring circulating vitamin C levels, is associated with lower risks of hypertension, coronary heart disease, and stroke [108]. This vitamin is also involved in the regeneration of fat-soluble vitamin E [109], which is particularly able to inhibit lipid peroxidation.

Naturally occurring vitamin E includes eight fat-soluble isoforms, but in the human body α-tocopherol is the most common isoform. Plant seeds, especially sunflower seeds, almonds, and hazelnuts are rich sources of α-tocopherol; moreover, many vegetable oils (e.g., olive oil and canola oil) and tomato, avocado, spinach, asparagus, Swiss chard, and broccoli also contain this vitamin. α-tocopherol is uniquely suited to intercept peroxyl radicals and thus prevent lipid peroxidation and the detrimental effects of free radicals in membranes and plasma lipoproteins [110]. α-tocopherol is also likely to be involved in cell-mediated immunity [111,112]. In addition to its direct antioxidant properties, vitamin E modulates signal transduction and gene expression in a redox-dependent and redox-independent manner, regulating cellular functions relevant for its action and for preventing a number of diseases, including cancer, atherosclerosis, inflammation, and neurodegenerative diseases [113]. Carotenoids are another important type of dietary antioxidant which play a protective role in many diseases. They are organic pigments present in fruits and vegetables like pumpkins, carrots, corn, and tomatoes [114]. They have important antioxidant functions such as singlet oxygen quenching and radical scavenging [115]. Recently, the benefits of carotenoids against oxidative stress in human have been reviewed [116]. Some carotenoids like beta-carotene are dietary precursors of the fat-soluble vitamin A, or retinol, with important antioxidant activity and liver protection functions [117]. In addition, vitamin A is converted in retinoic acid and functions as a ligand, regulating the expression of genes involved in cell metabolism [118]. Some evidence suggests that carotenoids beside their antioxidant activity also exert signaling functions. Indeed, carotenoids or their metabolites may up-regulate the expression of antioxidant or detoxifying enzymes via the activation of the Nrf2-dependent pathway [119].

Polyphenols are phytochemicals with antioxidant properties which occur in vegetal food [105]. Quercetin, a natural flavonoid found mainly in nuts, grapes, onions, broccoli, apples, and black tea, has shown important antioxidant activity and protective effects on the intestinal mucosal barrier [120], as well as the ability to reduce inflammation by suppressing the expression of pro-inflammatory mediators [121,122].

Resveratrol is a natural polyphenolic compound occurring in several plants and in food, including in red wine, peanuts, blueberries, raspberries, and mulberries [123]. In preclinical studies, it has been observed that resveratrol is important in the prevention and/or treatment of cancer, cardiovascular disease, and neurodegenerative diseases [124]. Another biologically active polyphenolic compound is curcumin, which is found in turmeric, a spice derived from the rhizomes of the plant *Curcuma longa* Linn. Many preclinical studies show that curcumin modulates numerous molecular targets and exerts antioxidant, anti-inflammatory, anticancer, and neuroprotective activities [125]. Furthermore, curcumin is important to prevent and treat Type 2 diabetes mellitus disease [126]. The effects of polyphenols cannot be explained solely on the basis of their antioxidant action; indeed, the health benefits of these substances may rely on their effects on enzyme, membrane, or nuclear receptors and intracellular transduction mechanisms [127]. Functions and sources of the main dietary antioxidants are summarized in Table 1.

In the elderly there is an impairment of the endogenous antioxidant defense system [141] and a decline of mitochondrial function associated with inadequate antioxidant dietary intake. Great interest has been devoted to antioxidant supplementation as a potential intervention in sarcopenia [142] since oxidative damage is considered to be one of the mechanisms leading to the loss of muscle mass and function.

Studies conducted in animal models in which supplementation of diet with antioxidants and physical activity have been combined in many cases seem to support the use of antioxidants to ameliorate exercise performance. Resveratrol, which is able to promote mitochondrial adaptive response and strength of upper limbs in mice after 12 weeks of treadmill exercise training suggests that this natural antioxidant could be used as a performance enhancer [138]. Resveratrol also exerts beneficial effects on muscle performance in animal models of aging. The dietary administration of resveratrol, in combination with habitual exercise in mice, has been shown to improve mitochondrial function and aging-related decline in physical performance [139]. In addition, curcumin supplementation ameliorates exercise performance in rats increasing time of run to exhaustion compared to control animals [137]. On the contrary, vitamin A administration in exercise training rats has been shown to induce lipid peroxidation and protein damage, decrease SOD1 levels, and attenuated exercise-dependent increase of SOD2 in the skeletal muscle [135].

However, the positive findings obtained in animals have not always been confirmed in human trials. In young football athletes, antioxidant supplementation has been shown to not counteract muscle damage or soreness induced by acute exercise and also to not ameliorate physical performance, although it has been seen to reduce oxidative stress [128]. Moreover, even where a decrease of oxidative stress or inflammatory markers and reduced onset of age-related pathophysiological disorders has been shown in human subjects assuming quercetin supplementation accompanied by constant and moderate physical exercise, an amelioration of muscle performance has not been clearly evidenced [132,133,134]. A recent review by Beaudart et al. [143] summarizing the results of 37 randomized clinical trials in which exercise and nutritional interventions, including the administration of natural antioxidants, were combined, evidenced a beneficial effect of exercise on muscle mass and strength in subjects over 65 years, while the effects of dietary antioxidant supplementation were more controversial. Moreover, importantly, some studies have highlighted that prolonged antioxidant supplementation can lead to undesirable effects like disruption of endogenous antioxidant levels, thus failing to counteract exercise-induced oxidative stress, and interfering with muscle adaptation to exercise [144,145,146]. Moreover, long-term administration of vitamin C has been observed to prevent mitochondrial biogenesis, decreasing the expression of endogenous antioxidant enzymes [131].

However, other literature data have suggested beneficial effects of antioxidant supplementation in muscle performance in normal young adult and sarcopenic subjects. A study conducted in subjects aged 65–80 years assuming resveratrol combined with 12 weeks of exercise has indicated a novel anabolic role of resveratrol in exercise-induced adaptations of older persons, and this suggests that this compound combined with exercise is likely to better counteract sarcopenia than exercise alone [140]. Moreover, the strongest evidence supporting the beneficial effects of antioxidant supplementation in physical performance has been obtained with vitamin E alone and in combination with vitamin C. Recently, He et al. [129] showed a decrease of markers of muscle damage, an amelioration of antioxidant status, and a delayed onset of muscle soreness after repeated downhill runs in moderately-trained males assuming vitamin C and E.

Several studies have reported the association between vitamin E and sarcopenia. In particular, Cesari et al. [130], in their study “Invecchiare in Chianti”, showed that vitamin E daily intake level positively correlated with knee extension strength and total physical performance. Moreover, it seems that vitamin C, by regenerating vitamin E, is responsible for muscle protection [147]. Flavonoids have many health benefits and improve exercise performance in athletes and in subjects not necessarily in constant training, such as aged people [148]. Finally, sarcopenia has also been associated with low levels of carotenoids even if further studies are necessary to better ascertain the existence of a direct link between low carotenoids status and muscle decline [136].

Several reasons could underlie these contradictory results. The lack of beneficial effects of antioxidants in sarcopenia could be due to the fact that supplemented antioxidants, which are non-enzymatic antioxidants, may not be able to make up for the enzymatic antioxidant deficiency that characterizes sarcopenia. It must also be considered that some antioxidant enzymes, such as SOD1 [149] and SOD2 [150], are up-regulated by ROS and that the scavenging effects of antioxidants could further down-regulate them, worsening muscle oxidative damage and performance.

Moreover, the dual role of ROS in muscle performance could explain the conflicting data obtained with different nutritional protocols and exercise settings. Antioxidant treatment associated with exercise could eliminate the adaptive response, and, therefore, it has been suggested that if antioxidants are administered before exercise-induced ROS levels reach their peak they can prevent the physiological function of ROS, while they can exert beneficial effects if administered after the bell-shaped curve of ROS has reached its summit [50].

The search for the right association of antioxidant supplementation in sarcopenic subjects who perform physical activity still has a long way to go. In experimental protocols considering the contemporary administration of antioxidant supplementation and physical training in elderly subjects, the type, strength, and duration of exercise, as well as the research design and the timing and extent of favorable effects of ROS in muscle adaptation to exercise [151] must be carefully taken into account in order to better clarify the issue.

## 7. Conclusions

**Figure 1:**
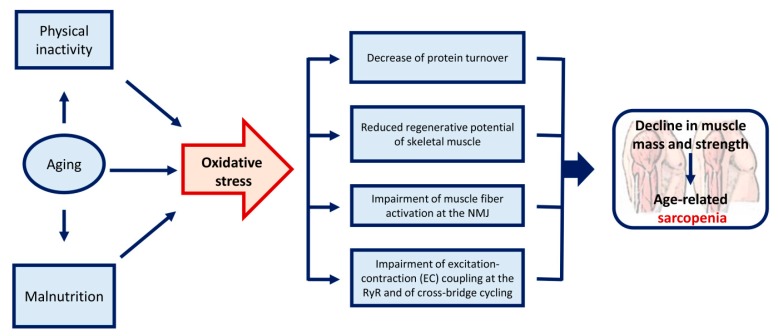
Aging, physical inactivity, and malnutrition lead to oxidative stress, contributing to sarcopenia.

Western societies are characterized by a progressive aging population which results in increasing prevalence of sarcopenia with subsequent increasing healthcare costs. Aging is associated with skeletal muscle oxidative stress due to increased ROS generation and impairment of antioxidant enzymes systems. On the other hand, muscle disuse and malnutrition are often associated with aging and also contribute to increased oxidative damage of skeletal muscle, leading to a decline in muscle mass and strength (Figure 1). From this complex scenario, it is clear that both physical activity and nutritional interventions must be contemplated for sarcopenic individuals.

Due to the central role played by oxidative stress in the onset of sarcopenia, a great effort has been focused on the understanding of the underlined cellular and molecular mechanisms involving ROS signaling in the attempt to identify the right strategies based on antioxidant supplementation for prevention and treatment of this condition.

Unfortunately, despite the fact that redox mechanisms leading to muscle mass and strength loss in sarcopenia have in part been unraveled, clinical data with antioxidant supplementation are far from clear and as a result of these conflicting reports, antioxidant supplementation cannot yet be considered as a nutritional intervention to prevent and treat sarcopenia. A more intense research effort must be made to clarify the right association between diet and exercise effective to counteract the onset and extent of age-related sarcopenia.

## Figures and Tables

**Table 1:** Functions and sources of principal nutritional antioxidants.

Nutritional Antioxidants	Functions	Sources	References Related to Effects on Skeletal Muscle
**Vitamin E (α–tocopherol)**	Boosts antioxidant defense Protects cell membranes Enhances immune functions Lipid peroxidation inhibitor	Vegetable oil, nuts, avocados	[128,129,130]
**Vitamin C (L-ascorbic acid)**	Principal hydrophilic antioxidant Scavenger of free radicals Enhances immune function Needed to make collagen, carnitine and neurotransmitters, e.g., serotonin and norepinephrine	Kiwifruit, strawberries, broccoli, kale, tomatoes, sweet red pepper	[128,129,131]
**Quercetin**	Antioxidant activity and protective effects on intestinal mucosal barrier Reduced exercise-induced lipid peroxidation Reduced oxidative stress and inflammatory biomarkers	Nuts, grapes, onions, broccoli, apples, black tea	[132,133,134]
**Carotenoids**	Scavenger of free radicals and singlet oxygen quenching Cancer prevention Prevention of liver diseases	Pumpkins, carrots, corn, tomatoes	[135,136]
**Curcumin**	Scavenger of reactive oxygen species (ROS) and reactive nitrogen species Neuroprotective activity Disease prevention (Type 2 diabetes mellitus, cancer)	*Curcuma longa*	[137]
**Resveratrol**	Prevention of cancer and cardiovascular disease Inhibition of neuroinflammation Increment of aerobic capacity	Skin of grapes, blueberries, raspberries, mulberries	[138,139,140]

## References

[1] Rosenberg I.H. (1997). Sarcopenia: Origins and clinical relevance. J. Nutr..

[2] Roubenoff R., Heymsfield S.B., Kehayias J.J., Cannon J.G., Rosenberg I.H. (1997). Standardization of nomenclature of body composition in weight loss. Am. J. Clin. Nutr..

[3] Cruz-Jentoft A.J., Baeyens J.P., Bauer J.M., Boirie Y., Cederholm T., Landi F., Martin F.C., Michel J.P., Rolland Y., Schneider S.M. (2010). Sarcopenia: European consensus on definition and diagnosis: Report of the European Working Group on Sarcopenia in Older People. Age Ageing.

[4] Cruz-Jentoft A.J., Sayer A.A. (2019). Sarcopenia. Lancet.

[5] Marques A., Queirós C., Hertz K., Santy-Tomlinson J. (2018). Frailty, Sarcopenia and Falls. Fragility Fracture Nursing: Holistic Care and Management of the Orthogeriatric Patient.

[6] Delbono O., O’Rourke K.S., Ettinger W.H. (1995). Excitation-calcium release uncoupling in aged single human skeletal muscle fibers. J. Membr. Biol..

[7] Wang Z.-M., Messi M.L., Delbono O. (2002). Sustained overexpression of IGF-1 prevents age-dependent decrease in charge movement and intracellular calcium in mouse skeletal muscle. Biophys. J..

[8] Brooks S.V., Faulkner J.A. (1994). Skeletal muscle weakness in old age: Underlying mechanisms. Med. Sci. Sports Exerc..

[9] Hook P., Li X., Sleep J., Hughes S., Larsson L. (1999). In vitro motility speed of slow myosin extracted from single soleus fibres from young and old rats. J. Physiol..

[10] Lexell J. (1995). Human aging, muscle mass, and fiber type composition. J. Gerontol. A Biol. Sci. Med. Sci..

[11] Frontera W.R., Suh D., Krivickas L.S., Hughes V.A., Goldstein R., Roubenoff R. (2000). Skeletal muscle fiber quality in older men and women. Am. J. Physiol. Cell Physiol..

[12] Joanisse S., Nederveen J.P., Snijders T., McKay B.R., Parise G. (2017). Skeletal Muscle Regeneration, Repair and Remodelling in Aging: The Importance of Muscle Stem Cells and Vascularization. Gerontology.

[13] Bohnert K.R., McMillan J.D., Kumar A. (2018). Emerging roles of ER stress and unfolded protein response pathways in skeletal muscle health and disease. Cell Physiol..

[14] Picca A., Fanelli F., Calvani R., Mulè G., Pesce V., Sisto A., Pantanelli C., Bernabei R., Landi F., Marzetti E. (2018). Gut Dysbiosis and muscle Aging: Searching for Novel Targets against Sarcopenia. Mediat. Inflamm..

[15] Dahlgren C., Karlsson A., Bylund J. (2019). Intracellular Neutrophil Oxidants: From Laboratory Curiosity to Clinical Reality. J. Immunol..

[16] Santillo M., Colantuoni A., Mondola P., Guida B., Damiano S. (2015). NOX signaling in molecular cardiovascular mechanisms involved in the blood pressure homeostasis. Front. Physiol..

[17] Xu H., Li C., Mozziconacci O., Zhu R., Xu Y., Tang Y., Chen R., Huang Y., Holzbeierlein J.M., Schöneich C. (2019). Xanthine oxidase-mediated oxidative stress promotes cancer cell-specific apoptosis. Free Radic. Biol. Med..

[18] Deby C., Goutier R. (1990). New perspectives on the biochemistry of superoxide anion and the efficiency of superoxide dismutase. Biochem. Pharmacol..

[19] Fridovich I. (1978). The biology of oxygen radicals. Science.

[20] McCord J.M., Fridovich I. (1996). Superoxide dismutase. An enzymic function for erythrocuprein (hemocuprein). J. Biol. Chem..

[21] Weisiger R.A., Fridovich I. (1973). Superoxide dismutase. Organelle specificity. J. Biol. Chem..

[22] Marklund S.L. (1982). Human copper-containing superoxide dismutase of high molecular weight. Proc. Natl. Acad. Sci. USA.

[23] Oldford C., Kuksal N., Gill R., Young A., Mailloux R.J. (2019). Estimation of the hydrogen peroxide producing capacities of liver and cardiac mitochondria isolated from C57BL/6N and C57BL/6J mice. Free Radic. Biol. Med..

[24] Del Río L.A., López-Huertas E. (2016). ROS Generation in Peroxisomes and its Role in Cell Signaling. Plant Cell Physiol..

[25] Formanowicz D., Radom M., Rybarczyk A., Formanowicz P. (2018). The role of Fenton reaction in ROS-induced toxicity underlying atherosclerosis—modeled and analyzed using a Petri net-based approach. Biosystems.

[26] Amodio G., Moltedo O., Faraonio R., Remondelli P. (2018). Targeting the Endoplasmic Reticulum Unfolded Protein Response to Counteract the Oxidative Stress-Induced Endothelial Dysfunction. Oxid. Med. Cell. Longev..

[27] Marciniak S.J., Yun C.Y., Oyadomari S., Novoa I., Zhang Y., Jungreis R., Nagata K., Harding H.P., Ron D. (2004). CHOP induces death by promoting protein synthesis and oxidation in the stressed endoplasmic reticulum. Genes Dev..

[28] Damiano S., Sasso A., Accetta R., Monda M., De Luca B., Pavone L.M., Belfiore A., Santillo M., Mondola P. (2018). Effect of Mutated Cu, Zn Superoxide Dismutase (SOD1G93A) on Modulation of Transductional Pathway Mediated by M1 Muscarinic Receptor in SK-N-BE and NSC-34 Cells. Front. Physiol..

[29] Potenza N., Mosca N., Mondola P., Damiano S., Russo A., De Felice B. (2018). Human miR-26a-5p regulates the glutamate transporter SLC1A1 (EAAT3) expression. Relevance in multiple sclerosis. Biochim. Biophys. Acta Mol. Basis Dis..

[30] Mondola P., Damiano S., Sasso A., Santillo M. (2016). The Cu, Zn Superoxide Dismutase: Not Only a Dismutase Enzyme. Front. Physiol..

[31] Accetta R., Damiano S., Morano A., Mondola P., Paternò R., Avvedimento E.V., Santillo M. (2016). Reactive Oxygen Species Derived from NOX3 and NOX5 Drive Differentiation of Human Oligodendrocytes. Front. Cell. Neurosci..

[32] Damiano S., Sasso A., De Felice B., Terrazzano G., Bresciamorra V., Carotenuto A., Orefice N.S., Orefice G., Vacca G., Belfiore A. (2015). The IFN-β 1b effect on Cu Zn superoxide dismutase (SOD1) in peripheral mononuclear blood cells of relapsing-remitting multiple sclerosis patients and in neuroblastoma SK-N-BE cells. Brain Res. Bull..

[33] Damiano S., Morano A., Ucci V., Accetta R., Mondola P., Paternò R., Avvedimento V.E., Santillo M. (2015). Dual oxidase 2 generated reactive oxygen species selectively mediate the induction of mucins by epidermal growth factor in enterocytes. Int. J. Biochem. Cell Biol..

[34] Damiano S., Petrozziello T., Ucci V., Amente S., Santillo M., Mondola P. (2013). Cu-Zn superoxide dismutase activates muscarinic acetylcholine M1 receptor pathway in neuroblastoma cells. Mol. Cell. Neurosci..

[35] Damiano S., Fusco R., Morano A., De Mizio M., Paternò R., De Rosa A., Spinelli R., Amente S., Frunzio R., Mondola P. (2012). Reactive oxygen species regulate the levels of dual oxidase (Duox1-2) in human neuroblastoma cells. PLoS ONE.

[36] Terrazzano G., Rubino V., Damiano S., Sasso A., Petrozziello T., Ucci V., Palatucci A.T., Giovazzino A., Santillo M., De Felice B. (2014). T cellactivation induces CuZnsuperoxide dismutase (SOD)-1 intracellular re-localization, production and secretion. Biochim. Biophys. Acta.

[37] Smith M.A., Reid M.B. (2006). Redox modulation of contractile function in respiratory and limb skeletal muscle. Respir. Physiol. Neurobiol..

[38] Debold E.P. (2015). Potential molecular mechanisms underlying muscle fatigue mediated by reactive oxygen and nitrogen species. Front. Physiol..

[39] Davies K.J., Quintanilha A.T., Brooks G.A., Packer L. (1982). Free radicals and tissue damage produced by exercise. Biochem. Biophys. Res. Commun..

[40] Urso M.L., Clarkson P.M. (2003). Oxidative stress, exercise, and antioxidant supplementation. Toxicology.

[41] Powers S.K., Jackson M.J. (2008). Exercise-induced oxidative stress: Cellular mechanisms and impact on muscle force production. Physiol. Rev..

[42] Ferreira L.F., Laitano O. (2016). Regulation of NADPH oxidases in skeletal muscle. Free Radic. Biol. Med..

[43] Cherednichenko G., Zima A.V., Feng W., Schaefer S., Blatter L.A., Pessah I.N. (2004). NADH oxidase activity of rat cardiac sarcoplasmic reticulum regulates calcium-induced calcium release. Circ. Res..

[44] Pearson T., Kabayo T., Ng R., Chamberlain J., McArdle A., Jackson M.J. (2014). Skeletal muscle contractions induce acute changes in cytosolic superoxide, but slower responses in mitochondrial superoxide and cellular hydrogen peroxide. PLoS ONE.

[45] Brandes R.P. (2005). Triggering mitochondrial radical release: A new function for NADPH oxidases. Hypertension.

[46] Zhang D.X., Chen Y.F., Campbell W.B., Zou A.P., Gross G.J., Li P.L. (2001). Characteristics and superoxide-induced activation of reconstituted myocardial mitochondrial ATP-sensitive potassium channels. Circ. Res..

[47] Gorlach A., Bertram K., Hudecova S., Krizanova O. (2015). Calcium and ROS: A mutual interplay. Redox Biol..

[48] Stamler J.S., Meissner G. (2001). Physiology of nitric oxide in skeletal muscle. Physiol. Rev..

[49] Ward C.W., Prosser B.L., Lederer W.J. (2014). Mechanical stretch-induced activation of ROS/RNS signaling in striated muscle. Antioxid. Redox Signal..

[50] Radak Z., Ishiharaa K., Tekusb E., Vargac C., Posac A., Baloghd L., Boldoghe I., Koltaia E. (2017). Exercise, oxidants, and antioxidants change the shape of the bell-shaped hormesis curve. Redox Biol..

[51] Oudot A., Martin C., Busseuilla D., Vergelya C., Demaisonc L., Rochette L. (2006). NADPH oxidases are in part responsible for increased cardiovascular superoxide production during aging. Free Radic. Biol. Med..

[52] Sullivan-Gunn M.J., Lewandowski P.A. (2013). Elevated hydrogen peroxide and decreased catalase and glutathione peroxidase protection are associated with aging sarcopenia. BMC Geriatr..

[53] Shi Y., Ivanikkov M.V., Walsh M.E., Liu Y., Zhang Y., Jaramillo C.A., Macleod G.T., Van Remmen H. (2014). The lack of CuZnSOD leads to impaired neurotransmitter release, neuromuscular junction destabilization and reduced muscle strength in mice. PLoS ONE.

[54] Muller F.L., Song W., Liu Y., Chaudhuri A., Pieke-Dahl S., Strong R., Huang T.T., Epstein C.J., Roberts L.J., Csete M. (2006). Absence of CuZn superoxide dismutase leads to elevated oxidative stress and acceleration of age-dependent skeletal muscle atrophy. Free Radic. Biol. Med..

[55] Fang C., Bourdette D., Banker G. (2012). Oxidative stress inhibits axonal transport: Implications for neurodegenerative diseases. Mol. Neurodegener..

[56] Liu Y., Chang A. (2008). Heat shock response relieves ER stress. EMBO J..

[57] Ivannikov M.V., Van Remmen H. (2015). Sod1 gene ablation in adult mice leads to physiological changes at the neuromuscular junction similar to changes that occur in old wild type mice. Free Radic. Biol. Med..

[58] Muller F.L., Song W., Jang Y.C., Liu Y., Sabia M., Richardson A., Van Remmen H. (2007). Denervation-induced skeletal muscle atrophy is associated with increased mitochondrial ROS production. Am. J. Physiol. Regul. Integr. Comp. Physiol..

[59] Watanabe M., Dykes–Hoberg M., Culotta V., Price D.L., Wong P.C., Rothstein J.D. (2001). Histological evidence of protein aggregation in mutant SOD1 transgenic mice and in amyotrophic lateral sclerosis neural tissues. Neurobiol. Dis..

[60] Okutsu M., Call J.A., Lira V.A., Zhang M., Donet J.A., French B.A., Martin K.S., Peirce-Cottler S.M., Rembold C.M., Annex B.H. (2014). Extracellular Superoxide Dismutase Ameliorates Skeletal Muscle Abnormalities, Cachexia and Exercise Intolerance in Mice with Congestive Heart Failure. Circ. Heart Fail..

[61] Mondola P., Ruggiero G., Serù R., Damiano S., Grimaldi S., Garbi C., Monda M., Greco D., Santillo M. (2003). The Cu, Zn superoxide dismutase in neuroblastoma SK-N-BE cells is exported by a microvesicles dependent pathway. Brain Res. Mol. Brain Res..

[62] Deldicque L. (2013). Endoplasmic reticulum stress in human skeletal muscle: Any contribution to sarcopenia?. Front. Physiol..

[63] Jheng J.R., Chen Y.S., Ao U.I., Chan D.C., Huang J.W., Hung K.Y., Tarng D.C., Chiang C.K. (2018). The double-edged sword of endoplasmic reticulum stress in uremic sarcopenia through myogenesis perturbation. J. Cachexia Sarcopenia Muscle.

[64] Bratic A., Larsson N.G. (2013). The role of mitochondria in aging. J. Clin. Investig..

[65] Sebastián D., Sorianello E., Segalés J., Irazoki A., Ruiz-Bonilla V., Sala D., Planet E., Berenguer-Llergo A., Muñoz J.P., Sánchez-Feutrie M. (2016). Mfn2 deficiency links age-related sarcopenia and impaired autophagy to activation of an adaptive mitophagy pathway. EMBO J..

[66] Tezze C., Romanello V., Desbats M.A., Fadini G.P., Albiero M., Favaro G., Ciciliot S., Soriano M.E., Morbidoni V., Cerqua C. (2017). Age-Associated Loss of OPA1 in Muscle Impacts Muscle Mass, Metabolic Homeostasis, Systemic Inflammation, and Epithelial Senescence. Cell Metab..

[67] Ji L.L., Zhang Y. (2014). Antioxidant and anti-inflammatory effects of exercise: Role of redox signaling. Free Radic. Res..

[68] Ghosh S., Karin M. (2002). Missing pieces in the NF-kappaB puzzle. Cell.

[69] Nader G., Esser K. (2001). Intracellular signaling specificity in skeletal muscle in response to different modes of exercise. J. Appl. Physiol..

[70] Chambers M.A., Moylan J.S., Smith J.D., Goodyear L.J., Reid M.B. (2009). Stretch-stimulated glucose uptake in skeletal muscle is mediated by reactive oxygen species and p38 MAP-kinase. J. Physiol..

[71] Thirupathi A., de Souza C.T. (2017). Multi-regulatory network of ROS: The interconnection of ROS, PGC-1 alpha, and AMPK-SIRT1 during exercise. J. Physiol. Biochem..

[72] Lin J., Wu H., Tarr P.T., Zhang C.Y., Wu Z., Boss O., Michael L.F., Puigserver P., Isotani E., Olson E.N. (2002). Transcriptional coactivator PGC-1 alpha drives the formation of slow-twitch muscle fibres. Nature.

[73] Gundersen K. (2011). Excitation-transcription coupling in skeletal muscle: The molecular pathways of exercise. Biol. Rev. Camb. Philos. Soc..

[74] Schiaffino S., Mammucari C. (2011). Regulation of skeletal muscle growth by the IGF1-Akt/PKB pathway: Insights from genetic models. Skelet. Muscle.

[75] Zhang J., Wang X., Vikash V., Ye Q., Wu D., Liu Y., Dong W. (2016). ROS and ROS-Mediated Cellular Signaling. Oxid. Med. Cell. Longev..

[76] Leslie N.R., Downes C.P. (2002). PTEN: The down side of PI 3-kinase signaling. Cell. Signal..

[77] Murata H., Ihara Y., Nakamura H., Yodoi J., Sumikawa K., Kondo T. (2003). Glutaredoxin exerts an antiapoptotic effect by regulating the redox state of Akt. J. Biol. Chem..

[78] Bassel-Duby R., Olson E.N. (2006). Signaling pathways in skeletal muscle remodeling. Annu. Rev. Biochem..

[79] Davies K.J.A. (2018). Cardiovascular Adaptive Homeostasis in Exercise. Front. Physiol..

[80] Santillo M., Pagliaro P. (2018). Editorial: Redox and Nitrosative Signaling in Cardiovascular System: From Physiological Response to Disease. Front. Physiol..

[81] St-Pierre J., Drori S., Uldry M., Silvaggi J.M., Rhee J., Jäger S., Handschin C., Zheng K., Lin J., Yang W. (2006). Suppression of reactive oxygen species and neurodegeneration by the PGC-1 transcriptional coactivators. Cell.

[82] Mason S.A., Morrison D., Mc Conell G.K., Wadley G.D. (2016). Muscle redox signaling pathways in exercise. Role of antioxidants. Free Radic. Biol. Med..

[83] Sakellariou G.K., Vasilaki A., Palomero J., Kayani A., Zibrik L., McArdle A., Jackson M. (2013). Studies of mitochondrial and nonmitochondrial sources implicate nicotinamide adenine dinucleotide phosphate oxidase(s) in the increased skeletal muscle superoxide generation that occurs during contractile activity. Antioxid. Redox Signal..

[84] Kang C., O’Moore K.M., Dickman J.R., Ji L.L. (2009). Exercise activation of muscle peroxisome proliferator-activated receptor-gamma coactivator-1alpha signaling is redox sensitive. Free Radic. Biol. Med..

[85] Ji L.L. (2015). Redox signaling in skeletal muscle: Role of aging and exercise. Adv. Physiol. Educ..

[86] Ebner N., Sliziuk V., Scherbakov N., Sandek A. (2015). Muscle wasting in ageing and chronic illness. ESC Heart Fail..

[87] Mosoni L., Malmezat T., Valluy M.C., Houlier M.L., Attaix D., Mirand P.P. (1999). Lower recovery of muscle protein lost during starvation in old rats despite a stimulation of protein synthesis. Am. J. Physiol..

[88] Rooyackers O.E., Adey D.B., Ades P.A., Nair K.S. (1996). Effect of age on in vivo rates of mitochondrial protein synthesis in human skeletal muscle. Proc. Natl. Acad. Sci. USA.

[89] Wenz T., Rossi S.G., Rotundo R.L., Spiegelman B.M., Moraes C.T. (2009). Increased muscle PGC-1alpha expression protects from sarcopenia and metabolic disease during aging. Proc. Natl. Acad. Sci. USA.

[90] Calvani R., Joseph A.M., Adhihetty P.J., Miccheli A., Bossola M., Leeuwenburgh C., Bernabei R., Marzetti E. (2013). Mitochondrial pathways in sarcopenia of aging and disuse muscle atrophy. Biol. Chem..

[91] Jackson M.J., McArdle A. (2016). Role of reactive oxygen species in age-related neuromuscular deficits. J. Physiol..

[92] Ji L.L. (2001). Exercise at old age: Does it increase or alleviate oxidative stress?. Ann. N. Y. Acad. Sci..

[93] Bejma J., Ji L.L. (1999). Aging and acute exercise enhances free radical generation and oxidative damage in skeletal muscle. J. Appl. Physiol..

[94] Derbré F., Gratas-Delamarche A., Gómez-Cabrera M.C., Viña J. (2014). Inactivity-induced oxidative stress: A central role in age-related sarcopenia?. Eur. J. Sport Sci..

[95] Bouzid M.A., Filaire E., Matran R., Robin S., Fabre C. (2018). Lifelong Voluntary Exercise Modulates Age-Related Changes in Oxidative Stress. Int. J. Sports Med..

[96] Tipton K.D. (2001). Muscle protein metabolism in the elderly: Influence of exercise and nutrition. Can. J. Appl. Physiol..

[97] Muscariello E., Nasti G., Siervo M., Di Maro M., Lapi D., D’Addio G., Colantuoni A. (2016). Dietary protein intake in sarcopenic obese older women. Clin. Interv. Aging.

[98] Vanacore D., Messina G., Lama S., Bitti G., Ambrosio P., Tenore G., Messina A., Monda V., Zappavigna S., Boccellino M. (2018). Effect of restriction vegan diet’s on muscle mass, oxidative status, and myocytes differentiation: A pilot study. J. Cell. Physiol..

[99] Monroy A., Lithgow G.J., Alavez S. (2013). Curcumin and neurodegenerative diseases. Biofactors.

[100] Pellavio G., Rui M., Caliogna L., Martino E., Gastaldi G., Collina S., Laforenza U. (2017). Regulation of Aquaporin Functional Properties Mediated by the Antioxidant Effects of Natural Compounds. Int. J. Mol. Sci..

[101] Boots A.W., Haenen G.R., Bast A. (2008). Health effects of quercetin: From antioxidant to nutraceutical. Eur. J. Pharmacol..

[102] Wu Q., Wang X., Nepovimova E., Wang Y., Yang H., Li L., Zhang X., Kuca K. (2017). Antioxidant agents against trichothecenes: New hints for oxidative stress treatment. Oncotarget.

[103] Damiano S., Sasso A., De Felice B., Di Gregorio I., La Rosa G., Lupoli G.A., Belfiore A., Mondola P., Santillo M. (2018). Quercetin Increases MUC2 and MUC5AC Gene Expression and Secretion in Intestinal Goblet Cell-Like LS174T via PLC/PKCα/ERK1-2 Pathway. Front. Physiol..

[104] Nieman D.C., Laupheimer M.W., Ranchordas M.K., Burke L.M., Stear S.J., Castell L.M. (2012). A-Z of nutritional supplements: Dietary supplements, sports nutrition foods and ergogenic aids for health and performance. Part 33. Br. J. Sport Med..

[105] Tresserra-Rimbau A., Arranz S., Vallverdu-Queralt A. (2017). New Insights into the Benefits of Polyphenols in Chronic Diseases. Oxid. Med. Cell. Longev..

[106] Fenech M., Amaya I., Valpuesta V., Botella M.A. (2018). Vitamin C Content in Fruits: Biosynthesis and Regulation. Front. Plant Sci..

[107] Levine M., Padayatty S.J., Ross A.C., Caballero B., Cousins R.J., Tucker K.L., Ziegler T.R. (2014). Modern Nutrition in Health and Disease.

[108] Hill A., Wendt S., Benstoem C., Neubauer C., Meybohm P., Langlois P., Adhikari N.K.J., Heyland D.K., Stoppe C. (2018). Vitamin C to Improve Organ Dysfunction in Cardiac Surgery Patients—Review and Pragmatic Approach. Nutrients.

[109] Cerullo F., Gambassi G., Cesari M. (2012). Rationale for Antioxidant Supplementation in Sarcopenia. J. Aging Res..

[110] Szymańska R., Nowicka B., Kruk J. (2017). Vitamin E—Occurrence, Biosynthesis by Plants and Functions in Human Nutrition. Mini Rev. Med. Chem..

[111] Marko M.G., Ahmed T., Bunnell S.C., Wu D., Chung H., Huber B.T., Meydani S.N. (2007). Age-associated decline in effective immune synapse formation of CD4(+) T cells is reversed by vitamin E supplementation. J. Immunol..

[112] Molano A., Meydani S.N. (2012). Vitamin E, signalosomes and gene expression in T cells. Mol. Asp. Med..

[113] Zingg J.M. (2019). Vitamin E: Regulatory Role on Signal Transduction. IUBMB Life.

[114] Elvira-Torales L.I., García-Alonso J., Periago-Castón M.J. (2019). Nutritional Importance of Carotenoids and Their Effect on Liver Health: A Review. Antioxidants.

[115] Sandmann G. (2019). Antioxidant Protection from UV- and Light-Stress Related to Carotenoid Structures. Antioxidants.

[116] Bohn T. (2019). Carotenoids and markers of oxidative stress in human observational studies and intervention trials: Implications for chronic diseases. Antioxidants.

[117] Yilmaz B., Sahin K., Bilen H., Bahcecioglu I.H., Bilir B., Ashraf S., Kucuk O. (2015). Carotenoids and non-alcoholic fatty liver disease. Hepatobiliary Surg. Nutr..

[118] Seif El-Din S.H., El-Lakkany N.M., El-Naggar A.A., Hammam O.A., Abd El-Latif H.A., Ain-Shoka A.A., Ebeid F.A. (2015). Effects of rosuvastatin and/or β-carotene on non-alcoholic fatty liver in rats. Res. Pharm. Sci..

[119] Kaulmann A., Bohn T. (2014). Carotenoids, inflammation, and oxidative stress—Implications of cellular signaling pathways and relation to chronic disease prevention. Nutr. Res..

[120] Kumar S., Pandey A.K. (2013). Chemistry and biological activities of flavonoids: An overview. Sci. World J..

[121] Espley R.V., Butts C.A., Laing W.A., Martell S., Smith H., McGhie T.K., Zhang J., Paturi G., Hedderley D., Bovy A. (2014). Dietary flavonoids from modified apple reduce inflammation markers and modulate gut microbiota in mice. J. Nutr..

[122] Lee S.G., Kim B., Yang Y., Pham T.X., Park Y.K., Manatou J., Koo S.I., Chun O.K., Lee J.Y. (2014). Berry anthocyanins suppress the expression and secretion of proinflammatory mediators in macrophages by inhibiting nuclear translocation of NF-kappaB independent of NRF2-mediated mechanism. J. Nutr. Biochem..

[123] Romero-Perez A.I., Ibern-Gomez M., Lamuela-Raventos R.M., de La Torre-Boronat M.C. (1999). Piceid, the major resveratrol derivative in grape juices. J. Agric. Food Chem..

[124] Aggarwal B.B., Bhardwaj A., Aggarwal R.S., Seeram N.P., Shishodia S., Takada Y. (2004). Role of resveratrol in prevention and therapy of cancer: Preclinical and clinical studies. Anticancer Res..

[125] Kuttan G., Kumar K.B., Guruvayoorappan C., Kuttan R. (2007). Antitumor, anti-invasion, and antimetastatic effects of curcumin. Adv. Exp. Med. Biol..

[126] Chuengsamarn S., Rattanamongkolgul S., Luechapudiporn R., Phisalaphong C., Jirawatnotai S. (2012). Curcumin extract for prevention of type 2 diabetes. Diabetes Care.

[127] Cipolletti M., Solar Fernandez V., Montalesi E., Marino M., Fiocchetti M. (2018). Beyond the Antioxidant Activity of Dietary Polyphenols in Cancer: The Modulation of Estrogen Receptors (ERs) Signaling. Int. J. Mol. Sci..

[128] De Oliveira D.C.X., Rosa F.T., Simões-Ambrósio L., Jordao A.A., Deminice R. (2019). Antioxidant vitamin supplementation prevents oxidative stress but does not enhance performance in young football athletes. Nutrition.

[129] He F., Hockemeyer J.A., Sedlock D. (2015). Does combined antioxidant vitamin supplementation blunt repeated bout effect?. Int. J. Sports Med..

[130] Cesari M., Pahor M., Bartali B., Cherubini A., Penninx B.W., Williams G.R., Atkinson H., Martin A., Guralnik J.M., Ferrucci L. (2004). Antioxidants and physical performance in elderly persons: The Invecchiare in Chianti (InCHIANTI) study. Am. J. Clin. Nutr..

[131] Gomez-Cabrera M.C., Domenech E., Romagnoli M., Arduini A., Borras C., Pallardo F.V., Sastre J., Viña J. (2008). Oral administration of vitamin C decreases muscle mitochondrial biogenesis and hampers training-induced adaptations in endurance performance. Am. J. Clin. Nutr..

[132] Simioni C., Zauli G., Martelli A.M., Vitale M., Sacchetti G., Gonelli A., Neri L.M. (2018). Oxidative stress: Role of physical exercise and antioxidant nutraceuticals in adulthood and aging. Oncotarget.

[133] Scholten S.D., Sergeev I.N. (2013). Long-term quercetin supplementation reduces lipid peroxidation but does not improve performance in endurance runners. Open Access J. Sports Med..

[134] McAnulty L.S., Miller L.E., Hosick P.A., Utter A.C., Quindry J.C., McAnulty S.R. (2013). Effect of resveratrol and quercetin supplementation on redox status and inflammation after exercise. Appl. Physiol. Nutr. Metab..

[135] Petiz L.L., Girardi C.S., Bortolin R.C., Kunzler A., Gasparotto J., Rabelo T.K., Matté C., Moreira J.C., Gelain D.P. (2007). Vitamin A Oral Supplementation Induces Oxidative Stress and Suppresses IL-10 and HSP70 in Skeletal Muscle of Trained Rats. Arch. Biochem. Biophys..

[136] Semba R.D., Lauretani F., Ferrucci L. (2007). Carotenoids as protection against sarcopenia in older adults. Arch. Biochem. Biophys..

[137] Sahin K., Pala R., Tuzcu M., Ozdemir O., Orhan C., Sahin N., Juturu V. (2016). Curcumin prevents muscle damage by regulating NF-kappaB and Nrf2 pathways and improves performance: An in vivo model. J. Inflamm. Res..

[138] Menzies K.J., Singh K., Saleem A., Hood D.A. (2013). Sirtuin 1-mediated effects of exercise and resveratrol on mitochondrial biogenesis. J. Biol. Chem..

[139] Murase T., Haramizu S., Ota N., Hase T. (2009). Suppression of the aging associated decline in physical performance by a combination of resveratrol intake and habitual exercise in senescence accelerated mice. Biogerontology.

[140] Always S.E., McCrory J.L., Kearcher K., Vickers A., Frear B., Gilleland D.L., Bonner D.E., Thomas J.M., Donley D.A., Lively M.W. (2017). Resveratrol Enhances Exercise-Induced Cellular and Functional Adaptations of Skeletal Muscle in Older Men and Women. J. Gerontol. A Biol. Sci. Med. Sci..

[141] Kregel K.C., Zhang H.J. (2007). An integrated view of oxidative stress in aging: Basic mechanisms, functional effects, and pathological considerations. Am. J. Physiol. Regul. Integr. Comp. Physiol..

[142] Kim J.S., Wilson J.M., Lee S.R. (2010). Dietary implications on mechanisms of sarcopenia: Roles of protein, amino acids and antioxidants. J. Nutr. Biochem..

[143] Beaudart C., Dawson A., Shaw S.C., Harvey N.C., Kanis J.A., Binkley N., Reginster J.Y., Chapurlat R., Chan D.C., Bruyère O. (2017). Nutrition and physical activity in the prevention and treatment of sarcopenia: Systematic review. Osteoporos. Int..

[144] Teixeira V.H., Valente H.F., Casal S.I., Marques A.F., Moreira P.A. (2009). Antioxidants do not prevent postexercise peroxidation and may delay muscle recovery. Med. Sci. Sports Exerc..

[145] Peternelj T.T., Coombes J.S. (2011). Antioxidant supplementation during exercise training: Beneficial or detrimental?. Sports Med..

[146] Rowlands D.S., Pearce E., Aboud A., Gillen J.B., Gibala M.J., Donato S., Waddington J.M., Green J.G., Tarnopolsky M.A. (2012). Oxidative stress, inflammation, and muscle soreness in an 894-km relay trail run. Eur. J. Appl. Physiol..

[147] Howard A.C., McNeil A.K., McNeil P.L. (2011). Promotion of plasma membrane repair by vitamin E. Nat. Commun..

[148] Fusco D., Colloca G., Lo Monaco M.R., Cesari M. (2007). Effects of antioxidant supplementation on the aging process. Clin. Interv. Aging.

[149] Mondola P., Annella T., Serù R., Santangelo F., Iossa S., Gioielli A., Santillo M. (1998). Secretion and increase of intracellular CuZn superoxide dismutase content in human neuroblastoma SK-N-BE cells subjected to oxidative stress. Brain Res. Bull..

[150] Selman C., McLaren J.S., Meyer C., Duncan J.S., Redman P., Collins A.R., Duthie G.G., Speakman J.R. (2006). Life-long vitamin C supplementation in combination with cold exposure does not affect oxidative damage or lifespan in mice, but decreases expression of antioxidant protection genes. Mech. Ageing Dev..

[151] Merry T.L., Ristow M. (2016). Do antioxidant supplements interfere with skeletal muscle adaptation to exercise training?. J. Physiol..

